# Place of death in patients with dementia and the association with comorbidities: a retrospective population-based observational study in Germany

**DOI:** 10.1186/s12904-018-0334-0

**Published:** 2018-05-24

**Authors:** Burkhard Dasch, Claudia Bausewein, Berend Feddersen

**Affiliations:** 1 Department of Anesthesiology, Intensive Care Medicine, Palliative Care Medicine and Pain Management, Berufsgenossenschaftliches Universitätsklinikum Bergmannsheil gGmbH Bochum, Medical Faculty of Ruhr University Bochum, Bürkle-de-la-Camp-Platz 1, 44789 Bochum, Germany; 2 Department of Palliative Medicine, Munich University Hospital, Ludwig-Maximilians- University Munich, Munich, Germany

**Keywords:** Dementia, Place of death, Comorbidities, Death certificate, Observational study, End-of-life care

## Abstract

**Background:**

Due to increasing life expectancy, more and more older people are suffering from dementia and comorbidities. To date, little information is available on place of death for dementia patients in Germany. In addition, the association of place of death and comorbidities is unknown.

**Methods:**

A population-based cross-sectional survey was conducted in Westphalia–Lippe (Germany), based on the analysis of death certificates from 2011. Individuals with dementia ≥ 65 years were identified using the documented cause of death. In this context, all mentioned causes of death were included. In addition, ten selected comorbidities were also analyzed. The results were presented descriptively. Using multivariate logistic regression, place of death was analyzed for any association with comorbidities.

**Results:**

A total of 10,364 death certificates were analyzed. Dementia was recorded in 1646 cases (15.9%; mean age 86.3 ± 6.9 years; 67.3% women). On average, 1.5 ± 1.0 selected comorbidities were present. Places of death were distributed as follows: home (19.9%), hospital (28.7%), palliative care unit (0.4%), nursing home (49.5%), hospice (0.9%), no details (0.7%). The death certificates documented cardiac failure in 43.6% of cases, pneumonia in 25.2%, and malignant tumour in 13.4%. An increased likelihood of dying in hospital compared to home or nursing home, respectively, was found for the following comorbidities (OR [95%-CI]): pneumonia (2.96 [2.01–4.35], *p* = 0.001); (2.38 [1.75–3.25], *p* = 0.001); renal failure (1.93 [1.26–2.97], *p* = 0.003); (1.65 [1.18–2.32], *p* = 0.003); and sepsis (13.73 [4.88–38.63], *p* = 0.001); (7.34 [4.21–12.78], *p* = 0.001).

**Conclusion:**

The most common place of death in patients with dementia is the retirement or nursing home, followed by hospital and home. Specific comorbidities, such as pneumonia or sepsis, correlated with an increased probability of dying in hospital.

## Background

The proportion of people developing a dementia-related disease increases with increasing age. Older people’s state of health is also usually characterized by comorbidity — i.e., they suffer from several diseases simultaneously.

In Germany, it is estimated that about 1.6 million people are currently diagnosed with dementia [1]. The absolute numbers of affected people have been estimated as 8.7 million in Europe in 2013 [2] and 46.8 million worldwide in 2015 [3]. Due to the age-dependency of the disease process and continually rising life expectancy, particularly in Western industrialized countries, the prevalence of the disease will increase further in the future. On the basis of predicted population trends in Germany, the number of patients with the condition will increase by around 40,000 annually and will rise to about 3 million by 2050 [1].

There is currently no treatment for dementia and the condition usually progresses very slowly. The duration of the disease cannot be reliably predicted in the individual case. Overall, the age-specific mortality rate is at least double that for individuals without dementia [4, 5]. Sampson et al. demonstrated in a prospective cohort of people with advanced dementia in the UK that 37% of these people died during a 9-months observational period [6]. Similar mortality rates have been reported in other countries [7–9].

During the course of the disease, people with severe dementia lose almost all their learned skills and abilities. They consequently require extensive nursing and medical support in many life situations. This represents a major health-policy and social challenge. It also affects end-of-life care. The disease is increasingly regarded as life-limiting by physicians, and the need for palliative care in patients in the advanced staged of dementia has been noted [10, 11].

Place of death is regarded as a kind of quality indicator for evaluating end-of-life care. Surveys on place of death show that most people clearly prefer to die at home rather than in institutions [12–14].

The place of death is not listed in official statistics in Germany, since the information given on the death certificate is not further analyzed by the relevant authorities. Studies on place of death for the general population in Germany show that hospitals are by far the most frequent place of death, followed by the home environment, retirement or nursing homes, hospices, and palliative care units [15].

Hardly any data regarding place of death are available for individuals with dementia in Germany. Escobar Pinzon et al. showed that in the federal state of Rhineland–Palatinate in 2008 42.4% of those with dementia died at home, followed by nursing homes (26.9%), hospitals (26.2%) and palliative institutions (hospices and/or palliative units; 3.2%) [16]. International studies show that individuals with dementia mainly die in institutions, with nursing homes and hospitals to some extent, being the most frequent place of death in most countries [16–22].

Older people with dementia often suffer from multiple additional diseases [23]. On average, two to eight other chronic diseases are present [24, 25]. In 3971 patients with dementia aged over 64 receiving care from family physicians in Spain, at least three other diagnoses were present in 70% with the most frequent being arterial hypertension, osteoarthrosis (in both women and men), as well as anxiety disorder/neurosis in women and benign prostate hypertrophy in men [26]. In the UK, arterial hypertension (53.4%), chronic pain (33.5%), depression (23.5%), presbyacusis (22.3%), coronary heart disease (21.6%), and chronic renal failure (20.8%) were the most frequent comorbidities in 4999 patients with dementia [27]. Overall, patients with dementia have a higher prevalence of complex situations that indicate functional limitations (including immobility, dysphagia, and impaired hearing), depression, and frailty syndrome (reduced physical activity, weakness, fatigue, weight loss) [28]. In addition, these patients have more often emergency hospital admissions compared to patients without dementia, and the number of hospital admissions increases with the severity of the disease [29, 30]. The reasons for hospital admission are often bronchial and urogenital infections, falls, or fractures, as well as delirium [31, 32]. Although it appears obvious from the clinical point of view that comorbidities contribute to the place of death for patients with dementia, hardly any scientific evidence is available on the topic.

The aim of the present study was to describe the place of death of patients with dementia in Germany on the basis of analyzed death certificates and to investigate the extent to which specific comorbidities are associated with the place of death.

## Methods

### Design

This was a population-based epidemiological cross-sectional study based on death certificates for the study region in 2011.

### Study region

The study region included selected urban areas (the cities of Bochum and Münster) and rural areas (the districts of Borken and Coesfeld) in Westphalia–Lippe in the federal state of North Rhine–Westphalia (Germany). On December 31st 2010, the study region’s population was 1,243,957, representing 1.5% of the total population of Germany at that time.

### Study data

The study used a complete dataset of death certificates for the study region. In all, 12,914 death certificates were available for 2011, which were archived in each local public health department and had to be analyzed on site due to data protection regulations. Information was collected about age, sex, time of death, place of death, manner of death, and cause of death. The main focus of the analysis was on the cause of death in patients with dementia. Ten other selected diseases documented by the physicians on the death certificate were also examined: pneumonia, aspiration, sepsis, cardiac failure, myocardial infarction, intracerebral bleeding (ICB) and/or cerebral stroke, malignant neoplasia, chronic obstructive pulmonary disease (COPD), renal failure, and Parkinson’s disease.

As cases of dementia mainly become clinically manifest in the elderly, the analyses was restricted to deceased persons whose age at death was 65 or over and who had a natural cause of death (*n* = 10,364).

### Documentation of cause of death

In accordance with German law, all deaths have to be certified by a physician. The form and structure of the death certificate are the responsibility of each federal state in Germany and are not standardized. In all 16 federal states, however, the question of the cause of death largely follows the scheme set out by the World Health Organization. Efforts have been made to develop a standard federal death certificate, but the project has so far been blocked by several states. It is also intended to introduce an electronic death certificate in Germany, as has been demanded at the European Union level, but this project has not yet been implemented [33].

The present study used death certificates from the state of North Rhine–Westphalia. Documentation of the cause of death is specified as follows here: “Section I,” I.a) “immediate cause of death” — i.e., the disease that led directly to death; I.b) “this is a result of” — i.e., a disease that is derived from the underlying condition and causally contributed to the death; I.c) “the underlying cause” — i.e., the disease causally leading to death and giving rise to the diseases described in I.a and I.b. In addition, the physician is able to record other diseases that were not immediately part of the causal chain leading to the death in “Section II.” The heading “Epicrisis” also provides an opportunity to document additional medical details on the sequence of the disease, accident occurrence, etc.

The analysis of death certificates is carried out in a standardized fashion in all federal states. The non-confidential section (time of death, manner of death, place of death) and the confidential section (cause of death) in the medical certificate are first sent to the local civil registry office where the patient was registered with his or her place of residence, and an official death statistic bulletin is drawn up. During this official procedure, the medical information about place of death is unfortunately not included. The death certificate is then sent on to the responsible public health office. There, the medical officer of health checks among other matters whether the stated diagnoses are compatible with the sex and age of the deceased and in general whether sufficient information about the cause of death is given. In a third step, the information is then transferred to the state statistical offices, where it is combined with the death statistic bulletin. Trained signatories once again check the medical details on the cause of death and finally determine the underlying disease in accordance with the regulations in the *International Statistical Classification of Diseases and Related Health Problems* (ICD), volume 2 [34]. This involves monocausal statistics on the cause of death — i.e., only one underlying disease is recorded and represented (“one cause per death”). The other diagnoses noted on the death certificate are ignored. Finally, this information is sent to the Federal Office of Statistics, which publishes annually cause-of-death statistics for the whole of Germany.

In contrast to the official cause-of-death statistics in Germany, the present study made use of all medical information available on the cause of death (Sections I.a, I.b, I.c, Section II, and epicrisis) in order to identify patients with dementia and other selected diseases. However, the medically documented diagnosis was not further differentiated according to Section I, Section II, or epicrisis. The reason for this was the highly time-consuming logistic effort involved in obtaining the documentation in each local public health office in the study region.

### Persons with dementia-related disease

In accordance with ICD-10, patients with a dementia-related disease constituted the study population if the medical details on the cause of death were described as follows: Alzheimer’s disease (F00, G30), vascular dementia (F01), dementia in other diseases classified elsewhere (F02), and unspecified dementia (F03).

### Comorbidities

All death certificates were analyzed for ten additional comorbid conditions and classified in accordance with ICD-10: pneumonia (J12.0–J18.9), aspiration (J69.0, J60.1, J69.8; J95.4; T17.2–T17.9), sepsis (A39.2–39.4, A40, A41, B37.7, R52.7), cardiac failure (I09, I25.1, I25.3–I25.9, I50), myocardial infarction (I21, I22, I24, I25.2), intracerebral bleeding (ICB) or cerebral stroke (I60, I61, I62, I63, I64, I69), malignant neoplasia (C00–C97), chronic obstructive pulmonary disease (COPD) (J41, J42, J44), renal failure (N17, N18, N19), and Parkinson’s disease (G20).

### Definition of place of death

The place of death was classified in the study as home environment, hospital, palliative care unit, retirement home or nursing home, hospice, and other locations. The category “home environment” combined the deceased person’s private residence as well as other private homes that were not the home of the deceased individual. Hospitals, psychiatric clinics, and sanatoriums were included under “hospital” as place of death. Palliative care units were counted as a separate place of death. The category “retirement or nursing home” included all institutions involving old age homes, retirement homes, geriatric care homes, sheltered housing, and short-term care. “Other locations” represented other public areas, family physicians’ practices, and leisure centers.

### Statistical analyses

To assess the prevalence, the absolute number of individuals aged 65 or over with a dementia-related disease was counted and related to the overall number of deaths in that age group (relative frequency). An analysis stratified by sex and specific age groups (65–69, 70–74, 75–79, 80–84, 85–89, 90–94, ≥ 95 years) was also carried out. In addition, the data were subjected to direct age standardization. For this purpose, the age-specific mortality rate in the study population was calculated, weighted with the age-specific rate in a standard population, and added up. The “Old European Standard Population” was used as the standard population.

The characteristics of the study population were listed by sex, age, selected comorbidities and number of comorbidities (no.: 1, 2, 3, 4, ≥ 5), and a subdivision relative to place of death was also carried out. It was investigated whether individuals who died at home with dementia differed significantly from those with a different place of death (hospital, palliative care unit, retirement or nursing home, hospice, other location, no details). For this purpose, unpaired *t*-tests were used for continuous data and the chi-squared test for categorical data, or in the case of cell numbers fewer than five, Fisher’s exact test was used.

Places of death were represented using absolute and relative frequencies, and sex-specific differences were tested using the chi-squared test. As no deaths at “other places” were observed, that category was not listed further in the results.

An association between “explanatory factors” and the dependent variable “place of death” was tested using a multivariate logic regression model. The target variable “home” (0) was investigated relative to the place of death “hospital” (1) and the place of death “retirement or nursing home” (1); in a second step, the place of death “retirement or nursing home” (0) was investigated relative to the places of death “hospital” (1). Due to very low results, the places of death “palliative care unit” (*n* = 6) and “hospice” (*n* = 14) were not subjected to multivariate regression analysis.

“Independent factors” were sex (women (1) vs. men (0)) and the median age of the deceased persons (≥ 86.7 y (1) vs. < 86.7 y (0)). In addition, the multivariate regression model considered all ten comorbidities — pneumonia, aspiration, sepsis, cardiac failure, myocardial infarction, intracerebral bleeding (ICB) or cerebral stroke, malignant tumour, chronic obstructive pulmonary disease (COPD), renal failure, and Parkinson’s disease. The process of modeling followed primarily clinical aspects. The aim was to analyze the statistical impact of each explanatory variable (sex, age, diseases) on the dependent variable “place of death”. Accordingly, we used a block method and not a stepwise regression procedure (forward selection or backward elimination). Under these conditions, we accepted a possibly poorer adjustment of the statistical model. Odds ratios with 95% confidence intervals were generated from this model. The Wald test was used to examine whether the independent variable had any significant influence on the target variable. The quality of the statistical model was expressed using Nagelkerke pseudo-*R*^2^ coefficients.

To minimize the global increase in the probability of alpha error due to multiple testing of the same sample, the significance level was set at *p* < 0.01 (two-sided). All analyses were carried out using the statistics program IBM SPSS Statistics, version 23.

### Ethics approval and data protection

The study was submitted to the Ethics Committee of the Ruhr University of Bochum and approved after examination (registry no. 4522-12). Letters were sent to the public health offices requesting access to the death certificates archived there. Permission to collect data and carry out the scientific analysis, while observing legal data protection regulations, was officially granted. The data had to be recorded locally in the public health offices.

## Results

**Fig. 1:**
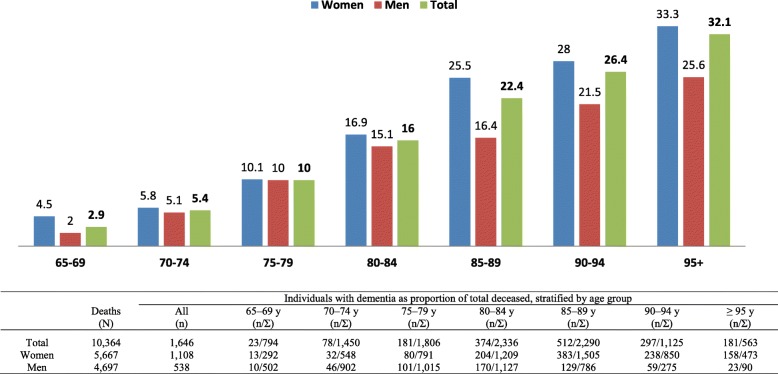
Prevalence of persons aged 65 or over with a death certificate recording dementia

A total of 10,364 death certificates of patients who had died at the age of 65 or over were analyzed. Dementia was described in 1646 cases, representing a relative frequency of 15.9%. A larger proportion of women (19.5%) than men (11.4%) were affected. The standardized prevalence of all individuals with a dementia-related disease was 8.0% (women 9.0%, men 6.9%; data not shown). One in ten deceased persons aged 75–79 suffered from dementia. In the 95 or older age group, one in four men and one in three women were affected by the disease (Fig. 1).

Overall, 67.3% of the deceased patients were women. Of deaths in retirement or nursing homes from dementia, two-thirds were women. Of deaths in hospital, just under half were men. The mean age was 86.3 ± 6.9 years. On average, 1.5 ± 1.0 other comorbidities were present. No comorbidity was present in 14.9% of cases, one additional condition was present in 40.6%, and three comorbidities were present in 10.6% of the cases. The most frequent accompanying disease was cardiac failure (43.6%). Pneumonia was documented in one-quarter of the cases, and cancer in just over one in ten. Patients with dementia who died in hospital suffered significantly more often from pneumonia, aspiration, sepsis, and renal failure compared to patients with dementia who died at home. Patients with dementia who died in hospices had malignant tumours more often compared to those who had received terminal care in the home environment (71.4% vs. 12.8%; *p* < 0.01) (Table 1).

**Table 1:** Characteristics of deceased persons with dementia aged 65 or over, stratified by place of death

	Overall (*n* = 1646)		Home (*n* = 327)		Hospital (*n* = 473)		Palliative care unit (*n* = 6)		Retirement or nursing home (*n* = 815)		Hospice (*n* = 14)		No details (*n* = 11)	
	%	n	%	n	%	n	%	n	%	n	%	n	%	n
Women	67.3	1108	64.5	211	55.4*	262	83.3	5	75.6*	616	42.9	6	72.7	8
Men	32.7	538	35.5	116	44.6*	211	16.7	1	24.4*	199	57.1	8	27.3	3
Age (mean / SD)	86.3 ± 6.9		85.8 ± 6.8		84.2* ± 6.5		81.5 ± 7.4		87.8* ± 6.8		81.1 ± 6.7		84.5 ± 5.4	
Age (median, 0.5 quantile)	86.7		86.4		84.6		84.9		88.2		81.9		83.9	
Age (0.25 quantile)	82.0		81.7		80.1		74.5		83.6		79.3		80.7	
Age (0.75 quantile)	90.7		90.4		88.9		86.7		92.0		85.5		86.7	
Age, women (mean / SD)	87.7 ± 6.6		87.2 ± 6.6		85.8 ± 6.4		80.7 ± 7.9		88.8* ± 6.4		82.6* ± 2.4		84.7 ± 6.3	
Age, men (mean / SD)	83.3 ± 6.5		83.4 ± 6.4		82.1 ± 6.0		85.5 ± 0		84.7 ± 6.8		80.0 ± 8.7		84.1 ± 2.9	
Pneumonia	25.2	415	19.3	63	39.1*	185	0	0	19.9	162	28.6	4	9.1	1
Aspiration	10.0	164	9.5	31	15.6*	74	0	0	7.1	58	7.1	1	0	0
Sepsis	5.7	93	1.2	4	14.4*	68	0	0	2.3	19	7.1	1	9.1	1
Cardiac failure	43.6	718	45.9	150	44.2	209	50.0	3	42.8	349	14.3	2	45.5	5
Myocardial infarction	4.8	79	4.6	15	7.2	34	0	0	3.7	30	0	0	0	0
ICB and/or cerebral stroke	13.0	214	13.1	43	10.8	51	16.7	1	14.2	116	14.3	2	9.1	1
Malignant tumour	13.4	220	12.8	42	13.3	63	33.3	2	12.3	100	71.4*	10	27.3	3
COPD	6.1	100	5.8	19	7.6	36	16.7	1	5.2	42	7.1	1	9.1	1
Renal failure	15.4	253	12.5	41	19.7*	93	33.3	2	14.4	117	0	0	0	0
Parkinson’s disease	9.4	154	8.0	26	11.4	54	16.7	1	9.0	73	0	0	0	0
Comorbidity (mean)	1.5 ± 1.0		1.3 ± 0.9		1.8 ± 1.0		1.7 ± 0.8		1.3 ± 0.9		1.5 ± 0.7		1.1 ± 0.8	
0 Comorbidity	14.9	245	16.8	55	5.9*	28	0.0	0	19.6	160	0.0	0	18.2	2
1 Comorbidity	40.6	669	45.3	148	33.4*	158	50.0	3	42.3	345	57.1	8	63.6	7
2 Comorbidities	31.1	512	27.8	91	39.1*	185	33.3	2	28.0	228	35.7	5	9.1	1
3 Comorbidities	10.6	175	8.6	28	15.9*	75	16.7	1	8.5	69	7.1	1	9.1	1
4 Comorbidities	2.2	36	1.5	5	4.9*	23	0.0	0	1.0	8	0.0	0	0.0	0
≥ 5 Comorbidities	0.5	9	0.0	0	0.8	4	0.0	0	0.6	5	0.0	0	0.0	0

*COPD* chronic obstructive pulmonary disease, *ICB* intracerebral bleeding, *SD* standard deviation

*Specific place of death vs. place of death “at home” (chi-squared test) *P* < 0.01

**Fig. 2:**
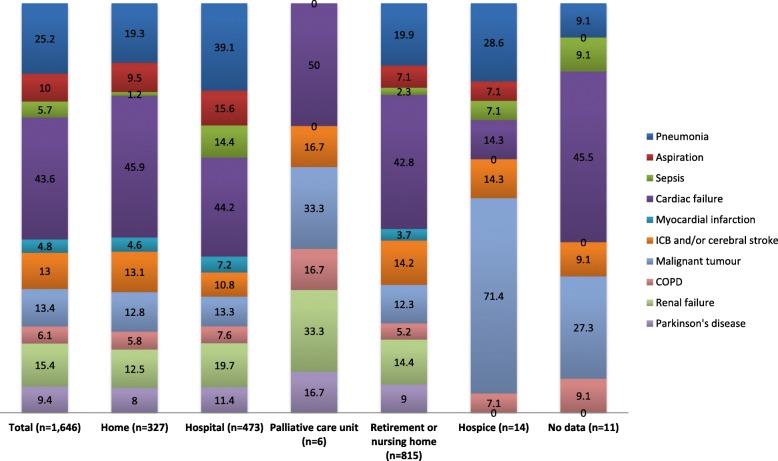
Specific comorbid conditions in persons aged 65 or over with dementia, stratified by place of death

Patients with dementia dying in hospital had a high proportion of infectious diseases (such as pneumonia or sepsis), aspiration, and renal failure in comparison with other places of death that were investigated. In contrast, septic conditions were only rarely noted in death certificates for those who died at home (1.2%), while cardiac failure was the most frequent in that location with 45.9%. In retirement or nursing homes, the frequency of documented pneumonia was similar to that for deaths in the home environment (19.9%) which was much lower in comparison with hospitals (39.1%). In hospices, dementia patients mainly died of tumours (Fig. 2).

**Fig. 3:**
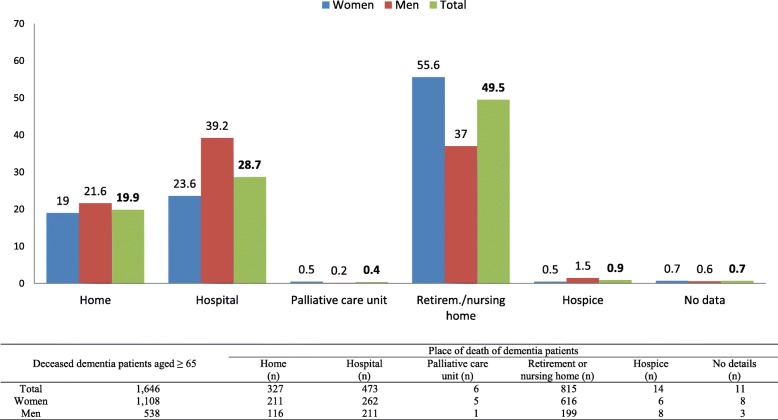
Place of death in persons aged 65 or over with dementia, stratified by gender

Retirement or nursing homes were by far the most frequent place of death. Approximately every second patient died there. Hospitals represented the second most frequent place of death (28.7%). Only one in five deaths occurred in the home environment. Palliative care units and hospices played a subordinate role, with a total of 1.3%. Stratified by sex, women died more often in retirement or nursing homes, while men by contrast died more often in hospital (Fig. 3).

In the multivariate regression analysis, very elderly patients with dementia (86 years, ≥ 0.5 quantile) and women had a higher odds of dying in retirement or nursing homes compared to the home setting (OR 1.59 [95%-CI 1.21–2.08], *p* = 0.001; OR 1.55 [95%-CI 1.16–2.08], *p* = 0.003) and a lower odds of dying in hospital compared to retirement or nursing homes (OR 0.50 [95%-CI 0.38–0.64], *p* = 0.001; OR 0.50 [95%-CI 0.38–0.66], *p* = 0.001). There was a statistical association between pneumonia, sepsis, and renal failure and hospitals as a place of death. In comparison with deaths at home, the odds of dying in hospital was three times higher when there was a medically documented pneumonia (OR 2.96 [95%-CI 2.01–4.35], *p* = 0.001), while in the presence of renal failure it was twice as high (OR 1.93 [95%-CI 1.26–2.97], *p* = 0.003) and with sepsis 14 times higher (OR 13.73 [95%-CI 4.88–38.63], *p* = 0.001). Similarly, in comparison with deaths in retirement or nursing homes, the odds of dying in hospital was also higher in the presence of pneumonia (OR 2.38 [95%-CI 1.75–3.25], *p* = 0.001), sepsis (OR 7.34 [95%-CI 4.21–12.78], *p* = 0.001) or renal failure (OR 1.65 [95%-CI 1.18–2.32], *p* = 0.003). There was also a correlation between the diagnosis of myocardial infarction and an increased probability of dying in hospital (OR 2.52 [95%-CI 1.45–4.36], *p* = 0.001) (Table 2).

**Table 2:** Association between place of death and specific comorbidities in persons aged 65 or over with dementia

	Hospital (1) vs. home (0)		Retirement or nursing home (1) vs. home (0)		Hospital (1) vs. retirement or nursing home (0)	
	OR (95% CI)	*p* value	OR (95% CI)	*p* value	OR (95% CI)	*p* value
Women (1) vs. men (0)	0.80 (0.58–1.11)	0.186	1.55 (1.16–2.08)*	0.003	0.50 (0.38–0.66)*	0.001
Age (median), ≥ 86.7 y (1) vs. < 86.7 y (0)	0.78 (0.57–1.08)	0.133	1.59 (1.21–2.08)*	0.001	0.50 (0.38–0.64)*	0.001
Pneumonia, yes (1) vs. no (0)	2.96 (2.01–4.35)*	0.001	1.25 (0.85–1.84)	0.249	2.38 (1.75–3.25)*	0.001
Aspiration, yes (1) vs. no (0)	1.26 (0.76–2.09)	0.380	0.66 (0.39–1.12)	0.126	1.68 (1.09–2.59)	0.018
Sepsis, yes (1) vs. no (0)	13.73 (4.88–38.63)*	0.001	1.71 (0.57–5.16)	0.338	7.34 (4.21–12.78)*	0.001
Cardiac failure, yes (1) vs. no (0)	1.29 (0.94–1.78)	0.113	0.82 (0.62–1.07)	0.145	1.51 (1.16–1.97)	0.012
Myocardial infarction, yes (1) vs. no (0)	2.19 (1.13–4.23)	0.019	0.86 (0.45–1.66)	0.661	2.52 (1.45–4.36)*	0.001
ICB /cerebral stroke, yes (1) vs. no (0)	1.01 (0.63–1.60)	0.984	1.10 (0.75–1.61)	0.642	0.87 (0.60–1.28)	0.471
Malignant tumour, yes (1) vs. no (0)	1.19 (0.75–1.87)	0.450	1.03 (0.69–1.53)	0.888	1.26 (0.87–1.83)	0.230
COPD, yes (1) vs. no (0)	1.28 (0.69–2.37)	0.439	0.98 (0.55–1.75)	0.957	1.42 (0.85–2.36)	0.182
Renal failure, yes (1) vs. no (0)	1.93 (1.26–2.97)*	0.003	1.15 (0.78–1.70)	0.483	1.65 (1.18–2.32)*	0.003
Parkinson’s disease, yes (1) vs. no (0)	1.39 (0.82–2.35)	0.227	1.20 (0.75–1.94)	0.449	1.12 (0.74–1.70)	0.577
Nagelkerke *R*^2^ (goodness of fit)	0.179		0.040		0.221	

*OR* odds ratio, *CI* confidence intervals, *COPD* chronic obstructive pulmonary disease, *ICB* intracerebral bleeding

*P < 0.01

## Discussion

Patients with dementia most often died in retirement or nursing homes, followed by hospitals and the home environment. Palliative care units and hospices as places of death played only a minor role. An association was seen between selected comorbidities and an increased likelihood of dying in hospital.

In the present study, nearly one in two deaths among patients with dementia occurred in retirement or nursing homes in Westphalia-Lippe (Germany). This finding is not surprising, as it reflects the high level of nursing care required by dementia patients. Relatives who are caring for people with dementia usually have a strong wish to care for and look after the patient in the shared home environment. However, relatives dealing with dementia patients on a daily basis are exposed to a large number of problems and challenges. The time demands involved in caring often conflict with the carer’s own family, and working life. Persons with dementia may also show depressive or even aggressive behavior in the course of their disease, as well as developing restlessness and/or a marked urge for movement. This can lead to a high level of physical and above all emotional burden on caring relatives, which may even cause social isolation [35, 36]. Caregivers usually belong to the patient’s immediate family (first-degree relatives, children, spouses), or more rarely they may be friends or other people linked to the patient [37]. If relatives struggle to cope with the situation they can get potentially support from a home help, a day or temporary nurse or a nursing service. However, these measures are often only effective in the shorter term. Alternative residential forms are available, such as “sheltered housing” or “dementia apartment-sharing,” but a move to a nursing home is often the only practicable way of ensuring care.

This study has shown that (unsurprisingly) it is mainly very elderly people and women who die in retirement and nursing homes. This observation is explained by demographic change and changes in social life. Life expectancy has been increasing for decades in the Western industrialized countries, and this applies to Germany as well. In this country, the mean life expectancy is currently 83.1 years for women and 78.2 years for men [38]. Due to their lower life expectancy, men are more likely to be survived by their partners, and this also increases the probability that they will be cared for by relatives at home at the end of their lives. Also, due to the increasing age, there is a greater likelihood that women will be widowed or living alone when they are elderly and, with increasing physical problems will be dependent on assistance from strangers or institutions. The results of the 2011 population census in Germany also indicate that more and more people are living alone. The proportion of people living alone, for example, increased from 15.6% in 1996 to 19.6% in 2011 [39].

Surveys have shown that most people would prefer to die at home [12–14] and this also applies to people with dementia-related diseases [16]. The findings of the present study are in contrast to this wish of patients: only one in five persons with dementia died at home. The reasons for this remain speculative, but it may again be linked to excessive stress on relatives caring for the patient and show that there is a need for relevant action to be taken in health care policy. This need has been recognized by the relevant German political decision-making body, and measures have been implemented [40]. The legal meaning of the term “status of requiring care” has been redefined and extended to mental and psychological illnesses. Patients with dementia have consequently had their previous benefit entitlement from the nursing insurance fund upgraded. In addition, relatives who have had to stop working in order to provide care are now receiving improved financial support from the state.

Advanced-stage dementia is increasingly being regarded as a terminal disease leading to death [10, 11, 41, 42]. Palliative care is appropriate in dementia, since it represents a “disease that does not respond to curative treatment” or a “life-threatening disease,” as dementia itself is not curable. The treatment approach aims at achieving improvements in quality of life. The results of the present study show that in-patient palliative and hospice institutions were only playing a minor role in 2011 in comparison with all other places of death. Only 0.4% of all dementia patients who died received end-of-life care in a palliative care unit, and only 0.9% of them received care in a hospice. There might be several reasons for this observation. First, a health economic aspect. There were only 32,1 palliative care unit beds and 40,2 hospice beds in the study region relative to a population of 1 million — corresponding to two-thirds of the maximum number recommended by the European Association for Palliative Care (EAPC) [43]. Thus, there was a need for implementing further inpatient palliative care and hospice services in this region in 2011. Second, the life expectancy of patients with dementia. In many cases, the natural course of the disease often exceeds the official requirements that patients should only be admitted to hospices when the medical estimate of life expectancy is less than 3–6 months.

Several investigations indicate that patients with dementia are at increased risk of hospital admission compared to people without dementia [29, 30, 43–46]. The reasons for this are complex [6, 31, 32, 47–49]. The most frequent causes include respiratory and urogenital infections, fall-related injuries, neurological and psychiatric causes (syncope, confusion, delirium), pressure sores, and nutritional disturbances. There is a consensus in the research findings that many of these diseases could have been treatable in home care or in in-patient care institutions, so that hospital admission could have been avoided [50, 51]. Psychosocial factors also affect hospital admissions — for example, when the previous carer suddenly becomes unavailable.

Generally, a hospital stay is a severe burden for many people with dementia and it is also associated with a number of risks. These include prolongation of the hospitalization period, a decline in physical functional abilities, increased frequencies of nosocomial infections, and an increased likelihood of not being able to return to the home environment after the hospital treatment [52]. Sampson et al. concluded that an unplanned hospital stay significantly shortens the median survival time in patients with dementia [53].

In the present study group of deceased individuals in the general population aged 65 or older with dementia, in-patient deaths represented 28.7% of cases. A similar percentage was reported by Houttekier et al. in a European survey in 2003 [18]. The mean percentage of dementia patients aged 65 or over who died in hospital in that study was 27.4%, including all countries investigated (Belgium, Netherlands, England, Wales, and Scotland). The Netherlands showed a very low percentage, with only 2.8% of deaths occurring in in-patients. In this country, some general practitioners (“verpleeghuisarts”) work exclusively in nursing homes enabling them to monitor the state of health of nursing-home patients tightly and offer medical treatment in a timely manner when physical changes occur. In most cases, hospital admissions can be avoided.

The medical information on cause of death that was analyzed in the present study showed that the deceased dementia patients had been suffering from a mean of 1.5 of the selected comorbidities. Cardiac failure was the most frequent comorbidity documented in the death certificates with almost one in two deaths, with the diagnoses of pneumonia, renal failure and malignant tumours following in frequency. Cardiovascular diseases are the most common cause of death in Germany, followed by cancers. The diagnosis of dementia is already in third place [54]. The prevalence of cardiac failure, like that of dementia, increases with increasing age [55, 56], and this may have contributed to the high prevalence of cardiac failure in the present sample. Many patients with advanced dementia also suffer from dysphagia [57], which may make fluid intake much more difficult. This can lead to dehydration and prerenal kidney failure. In addition, there is a risk of aspiration of fluid and food particles potentially resulting in pneumonia leading to sepsis and multiple-organ failure causing death finally [58]. In several autopsy studies, pneumonia was the most frequent cause of death [59, 60]. These findings are supported by clinical data. Mitchell et al. [7], for example, noted in the CASCADE study that 41.1% of the patients developed at least one episode of pulmonary infection during the 18-month follow-up period. The infection was associated with a high mortality rate. In the Netherlands, the three most frequent causes of death in nursing-home residents with dementia were dehydration (38%), cardiovascular diseases (19%), and respiratory infections (18%) [61]. In the present study, the death certificates described pneumonia in 25.2% of cases and aspiration in 10.0%. Pneumonia and/or aspiration were particularly frequent on the death certificates of dementia patients who died in hospital (39.1 and 15.6%, respectively). Compared to home or nursing home deaths, the odds of dying in hospital with documented pneumonia was two to three times higher, and with aspiration by a factor of 1.3 or 1.7 higher. In addition, deceased hospital patients often had sepsis (14.4%), renal failure (19.7%), and myocardial infarction (7.2%). These results suggest that there is a high intensity of treatment in hospital at the end of life in dementia patients. Unfortunately, the study was not able to provide any further information on this.

Care for patients with dementia in the last phase of their lives represents a special challenge, since those affected are often unable to express their treatment preferences directly themselves, while established, evidence-based treatment pathways for this phase of disease are still largely lacking. Research results showing that physical symptoms are widespread in persons with dementia and that they even increase before death [6, 7, 16, 61].

In Germany, a new law was passed in 2015 to improve hospice and palliative care [62]. The law aims to strengthen comprehensive hospice and palliative care in Germany by implementing targeted measures in statutory health insurance and social care insurance. The measures are intended among other things to ensure networking among medical and nursing services, as well as attendant hospice services, and to guarantee cooperation among the health-care providers involved. The aim is to strengthen palliative care and hospice approaches in in-patient care institutions and hospitals and to offer information to health-insurance policy-holders in a targeted way about the hospice and palliative care services available, as well as enabling nursing-home residents to carry out individualized care planning for the last phase of life. The statutory framework conditions have been set out, but they require specific arrangements and a financial basis so that everyone in Germany — and particularly those with dementia — can be offered adequate palliative medical care adapted to their individual needs at the end of their lives.

### Strengths and limitations

This study is based on the largest dataset (*n* = 10,364) analyzed to date on place of death in patients with dementia in Germany. No details were available regarding the place of death for only 0.7% of deceased persons with dementia aged 65 or over. As the study is related only to the selected study region of Westphalia–Lippe, the results are not representative of Germany as a whole.

The study design used a population-based cross-sectional survey. This methodological approach is very suitable for hypothesis generation, but it does not allow any causal conclusions to be drawn. The validity of such studies is also limited, since only a few variables are available for analysis. Important determining factors contributing to the place of death — such as the patient’s and/or relatives’ preference for place of death, marital status (single, married, divorced), residential situation (living alone or together with one or more other people), the amount of care required, information about treatments (chemotherapy, surgery, intensive-care procedures), links to a specialist team for palliative care, etc. — were not available for the analysis and could not be further explored for data protection reasons.

The medical details provided about the cause of death require critical reflection. For reasons of the logistics involved in obtaining the data, for example, this study did not differentiate among causes of death relative to the underlying disease, contributing factors and the final direct cause of death. On the other hand, for dementia and ten other diseases, it was possible to include all available information about the cause of death, which would otherwise not have been taken into account in the official statistics for cause of death. In consequence, the determined prevalence of dementia can be regarded as particularly reliable. The medical details about dementia given in the death certificates usually did not include either any information about the severity of the disease nor when it had started, so that in this respect no conclusions could be drawn. It should also be critically noted that the duration of the diseases investigated was not taken into account in any way in the recording and analysis of the data, since this information could not be accurately traced from the medical details.

It is known that dementia-related diseases are not always perceived by physicians as representing an underlying disease leading to death, and are consequently often not stated on death certificates [63]. This affects dementia patients who are being cared for at home more often than those in nursing homes. Due to this documentation practice, the frequency of dementia observed in the present study, particularly in the home environment, may be lower than is really the case.

The quality of the data given in death certificates is generally viewed critically. The form of the medical documentation contributes to this [33]. Illegible handwriting and varying choices of words to describe diagnoses often make it difficult to classify the medical details in accordance with ICD-10. A lack of knowledge on the part of physicians involved about the purpose of the details given (establishing a causal chain) also contributes to this. In addition, physicians often do not have any precise medical information about the deceased person’s clinical history. Without such knowledge, however, a precise cause of death can only be established with difficulty.

## Conclusion

The most common place of death in people with dementia was the retirement and nursing home, followed by hospital. Only one-fifth died in the home environment.

End-of-life care for people with dementia represents a special challenge and requires a person-centered care approach with staff qualified in palliative care. In this context, existing nursing and medical care services and hospice services need to be further developed and extended to ensure that all individuals with dementia can receive adequate palliative care in accordance with their own individual needs at the end of their lives.

## Abbreviations

CASCADE: Choices, Attitudes, and Strategies for Care of Advanced Dementia at the End-of-Life
COPD: Chronic obstructive pulmonary disease
EAPC: European Association for Palliative Care
ICB: Intracerebral bleeding
ICD: International Statistical Classification of Diseases and Related Health Problems
